# Entscheidungen treffen in Pandemiezeiten: Eine qualitative Studie zu Perspektiven von Leitungspersonen der stationären Langzeitpflege

**DOI:** 10.1007/s00391-022-02034-6

**Published:** 2022-02-24

**Authors:** A. Bieber, A. Dammermann, M. N. Dichter, C. Dinand, A. Eich-Krohm, S. Freytag, R. Möhler, M. Sander, R. Thalhammer, S. Fleischer

**Affiliations:** 1 Institut für Gesundheits- und Pflegewissenschaft, Medizinische Fakultät, Martin-Luther-Universität Halle-Wittenberg, Magdeburger Straße 8, 06112 Halle (Saale), Deutschland; 2 Institut für Sozialmedizin und Epidemiologie, Sektion für Forschung und Lehre in der Pflege, Universität zu Lübeck, Lübeck, Deutschland; 3 Institut für Pflegewissenschaft, Medizinische Fakultät und Uniklinik Köln, Universität zu Köln, Köln, Deutschland; 4 Fakultät für Gesundheit, Department für Pflegewissenschaft, Universität Witten-Herdecke, Witten-Herdecke, Deutschland; 5 Institut für Sozialmedizin und Gesundheitssystemforschung, Medizinische Fakultät, Otto-von-Guericke-Universität, Magdeburg, Deutschland; 6 Hochschule Fulda, Fulda, Deutschland; 7 Institut für Versorgungsforschung und Gesundheitsökonomie, Medizinische Fakultät, Universitätsklinikum Düsseldorf, Düsseldorf, Deutschland; 8 Fakultät für Angewandte Gesundheits- und Sozialwissenschaften, Technische Hochschule Rosenheim, Rosenheim, Deutschland

**Keywords:** Entscheiden, Corona, Krisen, Pflegeheime, Führungskräfte, To decide, Corona, Crisis, Long-term care facilities, Leaders

## Abstract

**Hintergrund:**

In der ersten Pandemiewelle im Frühjahr 2020 sind in den stationären Langzeitpflegeeinrichtungen überproportional viele Bewohner*innen und Mitarbeiter*innen an COVID-19 erkrankt und hatten den höchsten Anteil im Ausbruchsgeschehen. Leitungspersonen stationärer Altenpflegeeinrichtungen mussten pandemiebedingt teilweise täglich neue eigene Entscheidungen treffen sowie Entscheidungen übergeordneter Stellen interpretieren und integrieren.

**Ziel der Arbeit:**

Ziel war es zu beschreiben, welche Entscheidungen im Umgang mit der COVID-19-Pandemie von Leitungspersonen stationärer Altenpflegeeinrichtungen zu treffen waren, und welche Konsequenzen sich daraus ergaben.

**Material und Methoden:**

Es wurde ein qualitatives multizentrisches Querschnittdesign gewählt. Die Datenerhebung fand mittels semistrukturierter Telefoninterviews statt. Die aufgezeichneten Audiodaten wurden transkribiert, mittels Framework Analysis analysiert und in „peer debriefings“ reflektiert.

**Ergebnisse:**

Es konnten 78 Interviews in 43 Pflegeeinrichtungen geführt werden. Es wurden 3 Hauptthemen mit 10 Subthemen identifiziert: Entscheidungen zu sozialer Teilhabe; Entscheidungen zu Quarantäne und Isolation und Anpassen des Personaleinsatzes.

**Diskussion:**

Gebraucht werden klarere Information und Anordnungen zur Umsetzung von Maßnahmen, z. B. durch bundesweit einheitliche Vorgaben. In der Informationspolitik werden auch die Gesundheitsämter in der Pflicht gesehen. Konsequenzen ihrer Entscheidungen waren für die Leitungspersonen kaum absehbar und von Unsicherheit geprägt. Verantwortlichkeiten für und Konsequenzen von Entscheidungen in der Pandemie sollten weiter evaluiert werden, um Leitungspersonen für Krisenzeiten zu stärken.

**Zusatzmaterial online:**

Zusätzliche Informationen sind in der Online-Version dieses Artikels (10.1007/s00391-022-02034-6) enthalten.

## Abstract (en)

**Background:**

In the spring of 2020 during the first wave of the pandemic an above-average number of residents and staff in nursing homes fell ill and accounted for the highest number of incidences. Due to the pandemic, managers in nursing homes had to make new decisions on a daily basis as well as interpret and integrate decisions made by higher level authorities.

**Aim of the study:**

The aim was to describe the decisions that had to be made by the managers of nursing homes in dealing with the COVID-19 pandemic and related consequences.

**Material and methods:**

A qualitative multicentre cross-sectional design was chosen. Data collection was conducted with semi-structured telephone interviews. The recorded audio data were transcribed, analyzed using the framework analysis method and reflected in peer debriefings.

**Results:**

A total of 78 interviews were conducted in 43 nursing homes and 3 main themes with 10 subthemes emerged: decisions about social participation, decisions on quarantine and isolation and staff adjustments.

**Discussion:**

Clearer information and directives for the implementation of measures are needed, e.g. through standardized guidelines nationwide. Additionally, public health departments should play a stronger and more responsible role in a pandemic situation. The consequences of their decisions were hardly foreseeable for the managers and were marked by uncertainty. Responsibilities for and consequences of pandemic-related decisions should be further evaluated to empower managers in times of crises.

## Hintergrund und Zielsetzung/Fragestellung

In der ersten Pandemiewelle im Frühjahr 2020 sind in den stationären Langzeitpflegeeinrichtungen überproportional viele Bewohner*innen und Mitarbeiter*innen an COVID-19 erkrankt [24] und hatten den höchsten Anteil im Ausbruchsgeschehen [4]. Das Risiko von Bewohner*innen, an oder mit dem Virus zu versterben, war im Vergleich zu in der eigenen Häuslichkeit lebenden Gleichaltrigen deutlich erhöht [19, 21]. Gründe hierfür liegen zum einen in der höheren Komorbidität der in diesen Einrichtungen Lebenden, zum anderen aber auch in den durch räumliche und körperliche Nähe geprägten Lebensumständen [13, 21].

Dem Gesundheitsschutz stehen weitere Werte – soziale Teilhabe und Lebensqualität der Bewohner*innen – gegenüber. Eine Übersichtsarbeit zu psychosozialen Auswirkungen der Pandemie und Maßnahmen des Infektionsschutzes zeigt Einsamkeit, Trauer, Depressivität, aber auch Angst der Bewohner*innen als Folge von Kontakt- und Besuchsrestriktionen [2]. In den einbezogenen Studien wird auf die von Angehörigen berichtete Zunahme von Einsamkeit und reduzierter Lebensqualität verwiesen und die Angst von Mitarbeiter*innen vor Infektionen beschrieben. Aus geriatrischer Perspektive wird auf die Gefahr auch relativ kurzer Phasen intensiver sozialer Isolation für den Gesundheitszustand älterer Menschen hingewiesen [23]. Daraus ergibt sich die Forderung nach Infektionsschutzmaßnahmen, die so kurz wie möglich gehalten und deren Verhältnismäßigkeit stetig hinterfragt werden soll [23]. Das Prinzip der Verhältnismäßigkeit wird auch aus pflegewissenschaftlicher Perspektive betont, und dabei werden die Leitungspersonen vor Ort in der Verantwortung gesehen [5].

Leitungspersonen mussten pandemiebedingt teilweise täglich neue eigene Entscheidungen treffen sowie Vorgaben von übergeordneten Stellen interpretieren und umsetzen. Dabei mussten in der Kommunikation alle maßgeblichen Personengruppen berücksichtigt werden. In dieser Arbeit wird eine Entscheidung als Wahl einer Handlungsalternative definiert [12]. Bisher wurde jedoch nicht beschrieben, welche Entscheidungen im Umgang mit der COVID-19-Pandemie von Leitungspersonen stationärer Langzeitpflegeeinrichtungen zu treffen waren, und welche Konsequenzen sich daraus ergaben. Diese Lücke zu schließen, ist Ziel dieser Analyse.

## Studiendesign und Untersuchungsmethoden

Für die Studie wurde ein qualitatives multizentrisches Querschnittdesign mit semistrukturierten Telefoninterviews gewählt und in einem Verbund aus 10 Hochschulen in Deutschland und ihrer kooperierenden Einrichtungen bundesweit durchgeführt. Ein positives Votum der Ethikkommission der Martin-Luther-Universität Halle-Wittenberg lag vor (Nummer 2019-006 vom 25.05.2020).

Eingeschlossen wurden Leitungspersonen der administrativen Ebene bzw. der Wohnbereichsebene, um zum einen die Organisation und zum anderen die direkte Pflege und Versorgung berücksichtigen zu können. Der Kontakt zu den Teilnehmer*innen wurde über die kooperierenden Einrichtungen hergestellt. Für die Einrichtungs- und Pflegedienstleitungen sowie Wohnbereichsleitungen wurden separate Interview-Leitfäden verwendet (Zusatzmaterial online). Einrichtungscharakteristika und Merkmale der Studienteilnehmer*innen (Zusatzmaterial online) wurden separat erfasst. Die Interviews wurden in den Monaten Juni und Juli 2020 durchgeführt und digital aufgezeichnet.

Die Transkription erfolgte nach einheitlichen Transkriptionsregeln [6]. Die Daten wurden mittels Framework Analysis [20] unter Verwendung von MAXQDA 18 analysiert. Die Auswertungsmethode ermöglicht es, vorab festgelegte Themen, aber auch neue im Datenmaterial identifizierte Themen zu berücksichtigen [17]. Die Framework Analysis erfolgt in 5 Schritten: (1) „familiarization“ (vertraut machen), (2) „identifying a thematic framework“ (identifizieren wichtiger und wiederkehrender Themen), (3) „indexing“ (indexieren) (4) „charting“ (strukturierte Darstellung) und (5) „mapping and interpretation“ (Analyse) [7]. Für den 5. Analyseschritt (Mapping and interpretation) wurde das umfangreiche Datenmaterial 2 inhaltlichen Schwerpunkten zugeordnet und in „peer debriefings“ reflektiert. Der hier vorgestellte Schwerpunkt bezieht sich auf „Entscheidungen in der Pandemie“.

## Ergebnisse

### Stichprobe

Es wurden 43 stationäre Pflegeeinrichtungen in 10 Bundesländern in die Studie einbezogen. Befragt wurden 25 Einrichtungs- (EL), 15 Pflegedienst- (PDL) und 38 Wohnbereichsleitungen (WBL; Tab. 1). Die Interviews dauerten zwischen 20 und 101 min.

**Table 1:** 

Anzahl Interviewte		*n* = 78
Funktion	*Einrichtungsleitungen/Pflegedienstleitungen*	*n* = 40
	*Wohnbereichsleitungen*	*n* = 38
Berufserfahrung in der Funktion	*Einrichtungsleitungen/Pflegedienstleitungen*	
	≤ 2 Jahre	*n* = 10
	3–5 Jahre	*n* = 7
	6–9 Jahre	*n* = 5
	≥ 10 Jahre	*n* = 18
	*Wohnbereichsleitungen*	
	≤ 2 Jahre	*n* = 11
	3–5 Jahre	*n* = 2
	6–9 Jahre	*n* = 8
	≥ 10 Jahre	*n* = 17
Berufserfahrung, insgesamt	*Einrichtungsleitungen/Pflegedienstleitungen*	
	≤ 2 Jahre	*n* = 0
	3–5 Jahre	*n* = 2
	6–9 Jahre	*n* = 2
	≥ 10 Jahre	*n* = 35
	Keine Angabe	*n* = 1
	*Wohnbereichsleitungen*	
	≤ 2 Jahre	*n* = 0
	3–5 Jahre	*n* = 0
	6–9 Jahre	*n* = 5
	≥ 10 Jahre	*n* = 33
Erfahrungen mit Infektionskrankheiten	*Einrichtungsleitungen/Pflegedienstleitungen*	
	Ja	*n* = 40
	Nein	*n* = 0
	*Wohnbereichsleitungen*	
	Ja	*n* = 37
	Nein	*n* = 1

### Themen

Es konnten folgende 3 Hauptthemen identifiziert werden: Entscheidungen zu sozialer Teilhabe, Umsetzung von Quarantäne bzw. Isolation und Anpassen des Personaleinsatzes. Einen Überblick über die Themen und Subthemen mit Beschreibungen gibt Tab. 2.

**Table 2:** 

Themen	Subthemen
Entscheidungen zu sozialer Teilhabe	Entscheidungen hinsichtlich der Einschränkung persönlicher Kontakte
	Entscheidungen zum Umgang mit Bewohner*innen mit kognitiven Einschränkungen
	Entscheidungen in palliativen Situationen
	Entscheidungen zu Gruppenaktivitäten
Entscheidungen zu Quarantäne und Isolation	Nutzung von Räumlichkeiten
	Umzug/Verlegung von Bewohner*innen innerhalb der Einrichtung
	Einzug neuer Bewohner*innen
	Rückkehr von Bewohner*innen in die Einrichtung
Anpassen des Personaleinsatzes	Zuordnung von Aufgaben
	Veränderung des Arbeitsumfangs
	Änderung von Prozessen und Abläufen

#### Entscheidungen zu sozialer Teilhabe

Leitungspersonen mussten immer wieder neu entscheiden, ob, wann, mit wem, wie oft und wo Bewohner*innen mit anderen in Kontakt treten durften.

##### Entscheidungen hinsichtlich der Einschränkung persönlicher Kontakte.

Diesbezüglich wurden deutlich voneinander abweichende Entscheidungen berichtet, die sich auf alle Arten von Kontakten innerhalb der Einrichtung bezogen. Entweder es gab kaum Einschränkungen bzw. keine Regelungen, oder es wurden teils außergewöhnliche Entscheidungen getroffen, wie z. B. Sicherheitspersonal einzusetzen, um Personen zu hindern, die Einrichtung zu verlassen oder zu betreten. In einigen Einrichtungen kam es zu außergewöhnlichen Entscheidungen, wie die Errichtung eines Bauzauns rund um die Einrichtung oder gar der Einsatz von Stühlen zum Blockieren von Zimmertüren.

… mit einem Bauzaun um das Parkgelände der Einrichtung. So, dass wir wirklich jetzt eine bauliche Veränderung haben, die Ein- und Ausgänge sind zwar nicht geschlossen, aber das Gelände ist komplett umzäunt, da kommt keiner raus. (03_EL_01)

##### Entscheidungen zum Umgang mit Bewohner*innen mit kognitiven Einschränkungen.

Zum Umgang mit Bewohner*innen mit kognitiven Einschränkungen wurde insbesondere über die Schwierigkeiten berichtet, Entscheidungen zu Quarantäne/Isolierungsmaßnahmen durchzusetzen. Als nichtumsetzbar benannten einige Leitungspersonen die Maßnahmen für diese Personengruppen, während andere Leitungspersonen vom Ringen nach bewohnerbezogenen Entscheidungen berichten, wie Einzelfallregelungen in Absprache mit dem Gesundheitsamt.

Also der Umgang mit demenziell erkrankten Menschen, die unter Quarantäne sind. Das ist aus meiner Sicht überhaupt nicht geregelt. (09_WBL_01)

##### Entscheidungen in palliativen Situationen.

In palliativen Situationen wurde die Bedeutung von individuell, aber auch der Pandemie angemessenen Entscheidungen besonders deutlich. Leitungspersonen mussten dabei oft unmittelbar und ohne handlungsleitende Entscheidungsgrundlagen Festlegungen treffen. Die Widersprüche zwischen den Erfordernissen der sozialen Teilhabe und des Infektionsschutzes stellten Zerreißproben für die Leitungspersonen dar, wobei das Bestreben, eine Sterbebegleitung zu ermöglichen, gerade auch im Verdachts- oder Infektionsfall, prioritär angesehen wurde.

Sobald wir eine palliative Versorgung hatten, oder es lag jemand im Sterben, da durften die Angehörigen kommen und Abschied nehmen. (06_EL_06)

##### Entscheidungen zu Gruppenaktivitäten.

Leitungspersonen berichteten über in der Regel ausgesetzte Gruppenaktivitäten, mit Ausnahme von Einrichtungen, die nach dem Hausgemeinschaftskonzept arbeiten, wonach kleinere, feste Gruppen von Bewohner*innen den Alltag miteinander gestalten. Auch in diesen Einrichtungen wurde entschieden, wohnbereichsübergreifende Aktivitäten auszusetzen. Berichtet wurde über Entscheidungen zur Bildung von Kleingruppen, um soziales Leben für Bewohner*innen auch unter Pandemiebedingungen zu ermöglichen. Daneben gab es Einrichtungen, in denen generell nur Einzelbetreuung angeboten wurde.

Wir machen, seitdem wieder offen ist, Angebote in Kleingruppen mit 3 Leuten oder mit 5 Leuten. Die müssen dabei Mundschutz tragen oder sind draußen im Freien. Ansonsten macht unsere soziale Betreuung jetzt Einzelbetreuung. (06_WBL_05)

#### Entscheidungen zu Quarantäne und Isolation

Der Schutz der Bewohner*innen verlangte eine Reihe an Entscheidungen zur räumlichen und zur organisatorischen Umstrukturierung.

##### Nutzung von Räumlichkeiten.

Das Entscheidungsspektrum hinsichtlich der Nutzung von Räumlichkeiten reichte von keinen räumlichen Veränderungen, über die Umnutzung von Räumlichkeiten, wie pandemiebedingt nichtgenutzten Tagespflegeräumen, bis hin zur Einrichtung von Dreibettzimmern. Unterschiedliche Entscheidungen gab es hinsichtlich der Nutzung von Räumlichkeiten für die Mahlzeitenversorgung. Teilweise mussten Mahlzeiten ausschließlich in den Zimmern eingenommen werden; in anderen Einrichtungen wurde entschieden, nur einzelne Personen mit dem erforderlichen Abstand im Speisesaal zu versorgen. Die übliche Büfettform entfiel.

Und dann haben wir zum Anfang wirklich isoliert. Sodass die Mahlzeiten im Zimmer eingenommen wurden für sich alleine. (01_EL_01)

##### Umzug/Verlegung von Bewohner*innen innerhalb der Einrichtung.

Hinsichtlich der Verlegung/des Umzugs von Bewohner*innen wurden unterschiedliche Entscheidungen getroffen. In einigen Einrichtungen wurden Umzüge für erforderlich erachtet und auch umgesetzt, um Quarantänemöglichkeiten zu schaffen. In anderen Einrichtungen gab es Pläne für mögliche Verdachts- oder Infektionsfälle, die aufgrund ausgebliebener Fälle jedoch nicht umgesetzt wurden.

Wir haben teilweise wirklich Leute aus Einzelzimmern in ein Doppelzimmer verlegt, damit wir diese Einzelzimmer freihalten konnten, für die Quarantänezeit. (06_EL_06)

##### Einzug neuer Bewohner*innen.

Während in einigen Einrichtungen im Befragungszeitraum bewusst keine neuen Bewohner*innen aufgenommen wurden, entschieden Leitungspersonen anderer Einrichtungen, generell eine 14-tägige Quarantäne für neu eingezogene Bewohner*innen in der Einrichtung anzuwenden.

Erstmal durften wir ja keine neuen Bewohner aufnehmen und wenn neue Bewohner kommen, gehen die erstmal 14 Tage in Quarantäne. (02_EL_01)

##### Rückkehr von Bewohner*innen in die Einrichtung.

Bewohner*innen, die z. B. nach einem Krankenhausaufenthalt oder einem Spaziergang in die Einrichtung zurückkehren wollten, sahen sich je nach Einrichtung und Bundesland mit teilweise erheblich voneinander abweichenden Regelungen konfrontiert. Das Entscheidungsspektrum reichte hier von keinen Einschränkungen für Rückkehrende, über Testverfahren bis hin zu obligatorischer Quarantäne von unterschiedlicher Dauer zwischen 2 und 14 Tagen, z. T. je nach Bundesland in externen Einrichtungen, wie beispielsweise geschlossene Einrichtungen der Rehabilitation.

Also die (Bewohnerin) hat so ein Theater gemacht, sie durfte im Prinzip auch raus. Aber, sie musste immer anschließend in Quarantäne, weil sie einfach so resolut war, und hat sich nichts verbieten lassen. (07_WBL_01)

#### Anpassen des Personaleinsatzes

Um die Anforderungen bewältigen zu können, musste das Personal flexibel eingesetzt werden. Das wurde mitunter täglich neu entschieden.

##### Zuordnung von Aufgaben.

Zur Umsetzung notwendiger Kohortierung wurde über Entscheidungen berichtet, die Teams aufzuteilen, sodass ein Teil des Teams ausschließlich die Versorgung von Quarantäne- und Isolierungsfällen übernahm, während die anderen Mitarbeiter*innen für die Regelversorgung zuständig waren. Zum Teil wurde den Mitarbeiter*innen die Entscheidung überlassen, im Isolationsbereich zu arbeiten.

Der war vier, fünf Tage wirklich in der isolierten Versorgung. Eben dann nur (versorgt) durch die Fachkräfte. Das habe ich so entschieden. (05_WBL_03)

Berichtet wurde über notwendige Entscheidungen hinsichtlich der Erbringung von Leistungen neben der Pflege und Betreuung, wie Friseur- oder Einkaufsdienste. Aufgrund der Kontaktsperren für externe Dienstleister wurden solche Aufgaben z. T. zusätzlich von Mitarbeiter*innen der Einrichtungen übernommen.

##### Veränderung des Arbeitsumfangs.

Leitungspersonen berichteten über den deutlich erhöhten Arbeitsaufwand für ihren Aufgabenbereich, um notwendige Planungen und Entscheidungen zur Bewältigung der pandemiebedingten Herausforderungen durchsetzen zu können. Teilweise wurde entschieden, mehr Personal einzuplanen, um z. B. die aufwendigere Versorgung in den Zimmern abdecken zu können. Außergewöhnliche Entscheidungen waren u. a. der Einzug von Mitarbeiter*innen in die Einrichtung für einen definierten Zeitraum oder die Einführung von 12-h-Schichten auf freiwilliger Basis.

Aber ich war noch nie oder fühlte mich noch nie so in erster Reihe und in Verantwortung stehend, mit allen Konsequenzen. (06_EL_02)

##### Änderung von Prozessen und Abläufen.

Prozesse und Abläufe der Regelversorgung mussten den Pandemiebedingungen angepasst werden, was anhand konkreter Beispiele beschrieben wurde. Trotz allem versuchten viele Verantwortliche, die Situation der Bewohner*innen so erträglich wie möglich zu gestalten und Auswirkungen wie Vereinsamung durch die Isolation mit Maßnahmen entgegenzuwirken.

Haben die Betreuung auf den Wohnbereich komplett verlegt, vorrangig Einzelbetreuung, sodass wenig an Vereinsamung und Isolierung passiert ist. (05_EL_05)

## Diskussion

Leitungspersonen standen während der ersten Welle der COVID-19-Pandemie unter dem besonderen Druck, nahezu täglich Entscheidungen bezüglich des Infektionsschutzes treffen zu müssen und kreative Lösungen im Rahmen begrenzter und teilweise unklarer Handlungsalternativen zu finden. Diese Entscheidungen bezogen sich in unserer Befragung insbesondere auf Fragen zur sozialen Teilhabe innerhalb der Einrichtungen, auf die Umsetzung von Quarantäne bzw. Isolation und das Anpassen des Personaleinsatzes. Mit diesen Hauptthemen wird die Dialektik von Teilhabe und Gesundheitsschutz angesprochen sowie auf die Organisationsebene fokussiert. Ähnliche Herausforderungen für die Leitungsebene in Pflegeheimen wurden auch aus anderen Ländern berichtet [16]. Leitungspersonen mussten weitreichende Entscheidungen treffen, um Forderungen und Vorgaben erfüllen zu können. Die nur teilweise absehbaren Konsequenzen ihrer Entscheidungen mussten gegenüber Bewohner*innen und Mitarbeiter*innen und weiteren Beteiligten vertreten werden und trafen dabei auf z. T. konkurrierende Interessen. Eine Befragung von Leitungspersonen der stationären Langzeitpflege identifizierte den Umgang mit dem Infektionsrisiko als Kernthema der Herausforderungen, Belastungen und Bewältigungsstrategien der COVID-19-Pandemie [11]. Die in der vorliegenden Studie ermittelten Schwerpunkte der pandemiebedingten Entscheidungsprozesse unterstreichen dies. Gleichzeitig wurden dem Infektionsschutz zuwiderlaufende, aber für die Langzeitpflege bedeutsame Themen deutlich, wie die soziale Teilhabe der Bewohner*innen und eine personenzentrierte Werteorientierung insbesondere für Menschen mit Demenz. Dabei steht die Förderung von Selbstbestimmung und Lebensqualität auch unter Pandemiebedingungen im Fokus [5] und nimmt für Pflegeheimbewohner*innen mit depressiver Symptomatik einen hervorgehobene Stellung ein [22]. Die nach der ersten Welle erarbeitete Leitlinie zur Förderung sozialer Teilhabe und Lebensqualität in der stationären Altenpflege kann von Leitungspersonen als Entscheidungshilfe genutzt werden, um im weiteren Pandemieverlauf sicherer entscheiden zu können, z. B. hinsichtlich des Einschätzens individueller Risiken, des Zugangs zu bedarfsgerechten Angeboten der Gesundheitsversorgung oder der internen bzw. externen Kommunikation [5]. Ein weiteres bedeutsames Thema waren Entscheidungen im Zusammenhang mit palliativen Situationen. Leitungspersonen konnten diesbezügliche Entscheidungen nicht auf situationsgerechte Empfehlungen oder Richtlinien stützen, da diese kaum bzw. nicht verfügbar waren, wie aus Analysen internationaler Literatur hervorgeht [3, 9]. Die Versorgung Sterbender unter den Bedingungen der Kontakteinschränkungen wurde z. B. auch in den Niederlanden von den Pflegenden als problematisch und deutlich optimierbar beschrieben [14].

Bei der Umsetzung von Quarantäne und Isolation fielen notwendige Umnutzungen von Räumlichkeiten auf, um Kontakte reduzieren zu können. Einrichtungen, die nach dem sog. Hausgemeinschaftsmodell arbeiten, d. h. pro Wohnbereich ca. 12 Plätze vorhalten, scheinen im Vorteil zu sein, um trotz notwendiger Kontaktbeschränkung soziales Leben zu ermöglichen. Entsprechende Empfehlungen zu kleineren Wohnbereichen liegen vor, wie zum Konzept der Green Houses, die stärker auf den Alltag des Individuums eingehen als auf das Funktionieren einer Institution [1].

Für die Zukunft äußerten die Interviewten Befürchtungen bezüglich des Personaleinsatzes, u. a. dass erfahrene Mitarbeiter*innen als Reaktion auf die Pandemiekrise den Pflegeberuf verlassen könnten, eine Befürchtung, die sich mittlerweile national und international bestätigt hat [8, 18]. Gebraucht würden klarere Information und Anordnungen zur Umsetzung von Maßnahmen der Infektionsprävention und -kontrolle, z. B. durch bundesweit einheitliche Vorgaben; ein Wunsch, der nach wie vor nicht erfüllt ist. Bezüglich der Informationspolitik werden auch die Gesundheitsämter in der Pflicht gesehen. Die Verteilung von Schutzmaterialien sollte zentral gesteuert werden. Auf die Bedeutung einheitlicher Vorgaben und Richtlinien für Altenpflegeeinrichtungen wird auch in der internationalen Literatur verwiesen [15], als unverzichtbare Basis für einrichtungsspezifische Vorgaben der Infektionsprävention bzw. -kontrolle [10]. Für Einrichtungen ist zu fragen, wer für welche Entscheidungen Verantwortung übernehmen kann. So wird empfohlen, Pflegefachpersonen mehr Verantwortung zu übertragen, aber dafür auch entsprechend zu qualifizieren [10]. Es kann dabei nicht um simples Delegieren von Entscheidungen von einer zur anderen Ebene gehen, vielmehr ist einrichtungsübergreifend mit den Verantwortlichen der Gesundheitspolitik die Pandemie dahingehend auszuwerten, wer in Krisenzeiten welche Entscheidungen treffen sollte und welche Unterstützung dabei erforderlich ist.

### Stärken und Limitationen

Die Studie wurde als Multizenterstudie durchgeführt, mit dem Vorteil einer breiten Datenbasis aus einer Vielzahl von Pflegeeinrichtungen. Die Datenanalyse anhand der Framework Analysis ermöglichte die Identifikation von Themen zu Entscheidungsprozessen. Nicht Gegenstand dieser Analyse waren Entscheidungsprozesse, die neben den pandemiebezogenen Entscheidungen von den Leitungspersonen im Rahmen ihrer Routineaufgaben wahrgenommen wurden. Die Analyse ist in der Tiefe der Thematik Entscheidungsprozesse limitiert, da diese nicht originärer Bestandteil der Interview-Leitfäden waren. Eine weitere Limitation der Studie ist die Begrenztheit auf die erste Welle der COVID-19-Pandemie. Eine Fortsetzung der Studie zu den Erfahrungen aus den weiteren Pandemiewellen könnte die Thematik Umgang mit Entscheidungsprozessen explizit aufgreifen und an die hier vorgestellten Erkenntnisse anknüpfen. Die notwendigen Ressourcen müssten bereitgestellt werden, um nicht, wie in der vorliegenden Studie, komplett auf Eigenmittel der Projektpartner angewiesen zu sein.

## Fazit für die Praxis

- Leitungspersonen mussten in der ersten Pandemiewelle häufig weitreichende Entscheidungen treffen und wurden dabei unzureichend bzw. nicht unterstützt. Sie versuchten, eigenständig und verantwortungsbewusst bestmögliche Entscheidungen zu treffen. Die Förderung von Entscheidungskompetenz unter Krisenbedingungen sollte stärker in den Fokus der Fort- und Weiterbildung rücken.
- Die weiteren Wellen der Pandemie sollten dahingehend untersucht werden, welche Unterstützung Leitungspersonen stationärer Langzeitpflegeeinrichtungen in pandemiebedingten Entscheidungsprozessen erhielten bzw. benötigten. Weitere Entscheidungsträger sollten hierbei einbezogen werden, auch hinsichtlich der Bereitstellung von Ressourcen für Entscheidungsprozesse in Krisensituationen.
- Implikationen für die Praxis beziehen sich u. a. auf das Erstellen von Ablaufplänen und die Klärung von Zuständigkeiten, sowohl intern in der Pflegeeinrichtung als auch extern mit Netzwerkpartnern, Dienstleistern, Aufsichtsbehörden etc.
- Präventionsstrategien sind auf ihre Praxistauglichkeit und Angemessenheit zu überprüfen.

## Supplementary Information




