# Erratum zu: Entscheidungen treffen in Pandemiezeiten: Eine qualitative Studie zu Perspektiven von Leitungspersonen der stationären Langzeitpflege

**DOI:** 10.1007/s00391-022-02051-5

**Published:** 2022-03-10

**Authors:** A. Bieber, A. Dammermann, M. N. Dichter, C. Dinand, A. Eich-Krohm, S. Freytag, R. Möhler, M. Sander, R. Thalhammer, S. Fleischer

**Affiliations:** 1 Institut für Gesundheits- und Pflegewissenschaft, Medizinische Fakultät, Martin-Luther-Universität Halle-Wittenberg, Magdeburger Straße 8, 06112 Halle (Saale), Deutschland; 2 Institut für Sozialmedizin und Epidemiologie, Sektion für Forschung und Lehre in der Pflege, Universität zu Lübeck, Lübeck, Deutschland; 3 Institut für Pflegewissenschaft, Medizinische Fakultät und Uniklinik Köln, Universität zu Köln, Köln, Deutschland; 4 Fakultät für Gesundheit, Department für Pflegewissenschaft, Universität Witten-Herdecke, Witten-Herdecke, Deutschland; 5 Institut für Sozialmedizin und Gesundheitssystemforschung, Medizinische Fakultät, Otto-von-Guericke-Universität, Magdeburg, Deutschland; 6 Hochschule Fulda, Fulda, Deutschland; 7 Institut für Versorgungsforschung und Gesundheitsökonomie, Medizinische Fakultät, Universitätsklinikum Düsseldorf, Düsseldorf, Deutschland; 8 Fakultät für Angewandte Gesundheits- und Sozialwissenschaften, Technische Hochschule Rosenheim, Rosenheim, Deutschland

**Erratum zu:**

**Z Gerontol Geriat 2022**

10.1007/s00391-022-02034-6

Der Name des Autors M. N. Dichter war zunächst unvollständig. Der Originalbeitrag wurde korrigiert.

