# Supplementary material for: Making decisions in times of a pandemic: A qualitative study on perspectives of managers of long-term care facilities
Source: Z Gerontol Geriatr. 2022 Feb 24;55(2):143–9. [Article in German] doi: 10.1007/s00391-022-02034-6 (PMC8867686; doi:10.1007/s00391-022-02034-6)
Supplement: Supplementary file 1 [file 391_2022_2034_MOESM1_ESM.docx]

**HEICO -** Pflege**hei**me in der **CO**VID-19-Pandemie

Interviewleitfaden Einrichtungsleitungen

| **Die COVID-19-Pandemie stellt stationäre Altenpflegeinrichtungen vor enorme Herausforderungen. Mit diesem Interview wir herausfinden, , wie Sie die Situation in ihrer Einrichtung unter den Bedingungen der COVID-19-Pandemie wahrgenommen haben.** | | |
| --- | --- | --- |
| **Nr.** | **Thema/Fragen** | **Mögliche Nachfragen** |
| 1. | **Eingangsfrage:**  **Ich möchte gerne von Ihnen erfahren, wie Sie als Einrichtungsleitung die besondere Situation in Ihrer Einrichtung unter den Bedingungen der COVID-19 Pandemie wahrgenommen haben und jetzt wahrnehmen?** | - Je nach Antworten.   - Belastung (eigene, MA, Bew, Ang.)   - Vorgaben   - Unterstützung |
| 2. | **Bitte erzählen Sie mir, wie sich die Situation Ihrer Einrichtung im Verlauf der Pandemie entwickelt hat, von Beginn der Pandemie über die Phase der Kontaktbeschränkung hin zur allmählichen Öffnung?** | - Bewohner*innen - Team - Angehörige |
| 3. | **Bitte erzählen Sie mir von der Umsetzung des Pandemieplans in Ihrer Einrichtung und Schwierigkeiten, die dabei aufgetreten sind.** | - Gibt es einen Krisenstab in der Einrichtung? - Eventuell auch projektübergreifender Krisenstab - Die Organisation betreffend (z. B. Besuchsverbote, Kontaktbeschränkungen)? - Die direkte Pflege und Versorgung betreffend? - Den Personaleinsatz betreffend? - Den Einsatz von ehrenamtlich Aktiven betreffend? - Hygienevorgaben, Beschaffungswesen, Kooperation mit Therapeut*innen und Haus- bzw. Fachärzt*innen, etc.) betreffend? |
| 4. | **Bitte erzählen Sie mir, wie die Einhaltung der Vorgaben zur Kohortierung/Isolierung bei Ihnen umgesetzt werden konnte.** | - Bewohner*innen? - Mitarbeiter*innen? - Bauliche Veränderungen? |
| 5. | **Wie haben sich die Maßnahmen zur Kontaktbeschränkung auf die sozialen Aktivitäten, wie gemeinsames Essen, Veranstaltungen oder Gruppenangebote ausgewirkt?** | - Einzel- /Gruppenangebote? - Essen in Gemeinschaftsräumen? - Veranstaltungen? - Personal (z.B. mehr Stunden?) |
| 6. | **Bitte erzählen Sie mir von Maßnahmen, mit denen Sie versucht haben und aktuell versuchen, die soziale Teilhabe und die Lebensqualität der Bewohner*innen trotzdem aufrechtzuerhalten.** | - Digitale Lösungen? - Besuchsregelungen für Ärzt*innen, Therapeut*innen, Angehörige anderer Berufsgruppen, Angehörige, Freunde |
| 7. | **Bitte erzählen Sie mir, wie Sie über die Pandemie und die daraus resultierenden Maßnahmen innerhalb der Einrichtung kommuniziert haben?** | - Bewohner*innen? - Mitarbeiter*innen? |
| 8. | **Bitte erzählen Sie mir, wie Sie über die Pandemie und die daraus resultierenden Maßnahmen nach außen kommuniziert haben?** | - Angehörige, gesetzliche Betreuer? - Ärzt*innen, Therapeut*innen, etc.? - Gesundheitsamt? - Kommune? - Träger? |
| 9. | **Wir gehen davon aus, dass die Pandemie mit einem erheblichen Mehraufwand für Sie verbunden war. Bitte erzählen Sie mir, wie das genau war.** | - Mehraufwand im Personalmanagement? - Schulung von Mitarbeiter*innen? - In der Kommunikation mit Angehörigen? - In der Kommunikation mit externen Akteuren (z.B. Gesundheitsamt? Ärzt*innen?, Therapeut*innen?) - In der internen Kommunikation? - Im Beschaffungswesen? - Was bedeutet die Pandemie für Ihre Einrichtung finanziell? |
| 10. | **Standen und stehen Ihnen bzw. Ihren Mitarbeiter*innen ausreichend persönliche Schutzausrüstungen und Desinfektionsmittel zur Verfügung?** | - Wie häufig nicht? - Was hat gefehlt? - Worüber beschaffen Sie Material? - Ist die Unterstützung durch öffentliche Stellen hilfreich? |
| 11. | **Gibt es aktuell ein Thema, das Ihnen Probleme oder Sorgen im Zusammenhang mit Ihrer Einrichtung und der COVID-19 Pandemie bereitet?** | - Aktuelle Situation, Vorgehen Besuchsregelung |
| 12. | **Wo sehen Sie dringenden Unterstützungsbedarf im Zusammenhang mit der COVID-19 Pandemie für Ihre Einrichtung oder generell für Einrichtungen der stationären Altenpflege?** |  |
| 13. | **Überleitung zum Themenkomplex Gewalt und Gewaltprävention:**  **Wir können uns vorstellen, dass die COVID-19-Krise zu Stress und vielleicht auch Spannungen zwischen Pflegekräften und Bewohner*innen führen kann, die nicht immer gut zu lösen sind.** | |
|  | Wenn Sie an die aktuellen Herausforderungen der COVID-19 Krise denken und an die Auswirkungen auf die Bewohner*innen und Mitarbeiter*innen in ihrer Einrichtung, welche Bedeutung hat für Sie das Thema Prävention von Gewalt? |  |
| 14. | **Auswirkung der COVID-19 Pandemie auf das Gewaltgeschehen in den Einrichtungen** | |
|  | - Inwiefern hat sich nach Ihrer Einschätzung das Gewaltgeschehen in Ihrer Einrichtung durch die aktuellen Herausforderungen der Pandemie verändert? - Erzählen Sie bitte von einer Situation im Zusammenhang mit Gewalt, in der Sie oder Ihre Kolleg*innen dachten, das wäre ohne COVID-19 nicht passiert? |  |
| 15. | **Maßnahmen zur Gewaltprävention** | |
|  | - Welche Maßnahmen zur Gewaltprävention werden in Ihrer Einrichtung generell umgesetzt? - Welche Maßnahmen würden Sie als sinnvoll erachten, um dem Thema Gewalt in der aktuellen Situation zu begegnen? |  |
| 16. | **Umsetzbarkeit der Maßnahmen zur Gewaltprävention unter den aktuellen Herausforderungen der COVID19 Pandemie** | |
|  | - Was denken Sie, wie gut sind die Maßnahmen zur Gewaltprävention unter den aktuellen Herausforderungen [der COVID-19 Pandemie] umsetzbar? |  |
| **Ergänzende Frage für PEKo teilnehmende Einrichtungen:** | | |
| 17. | **Profit/ Mehrwert durch die Teilnahme am Projekt PEKo bzw. die geplanten und umgesetzten Maßnahmen zur Gewaltprävention** | |
|  | - Erzählen Sie bitte von einer Situation im Zusammenhang mit COVID-19, in der Sie dachten, gut, dass wir bei PEKo mitgemacht haben? - Wenn Sie sich zurückerinnern und vergleichen: Sind Sie aus Ihrer Sicht in der aktuellen Situation durch die im PEKo-Projekt geplanten und umgesetzten Maßnahmen zur Gewaltprävention besser aufgestellt? |  |

**HEICO -** Pflege**hei**me in der **CO**VID-19 Pandemie

Interviewleitfaden Wohnbereichsleitungen

| **Die COVID-19 Pandemie stellt stationäre Altenpflegeinrichtungen vor enorme Herausforderungen. Mit diesem Interview wir herausfinden, , wie Sie die Situation in ihrer Einrichtung unter den Bedingungen der COVID-19 Pandemie wahrgenommen haben.** | | |
| --- | --- | --- |
| **Nr.** | **Thema/Fragen** | **Mögliche Nachfragen** |
| 1. | **Eingangsfrage:**  **Ich möchte gerne von Ihnen erfahren wie Sie als Wohnbereichsleitung die besondere Situation in Ihrer Einrichtung unter den Bedingungen der COVID-19 Pandemie wahrgenommen haben und jetzt wahrnehmen?** | - Je nach Antworten |
| 2. | **Bitte erzählen Sie mir, wie sich die Situation ihres Wohnbereichs im Verlauf der Pandemie entwickelt hat, von Beginn der Pandemie über die Phase der Kontaktbeschränkung hin zur allmählichen Öffnung?** | - Bewohner*innen - Team - Angehörige |
| 3. | **Bitte erzählen Sie mir welche Veränderungen haben Sie auf Ihrem Wohnbereich aufgrund der Pandemie und der daraus resultierenden Vorgaben wahrgenommen?** | - Die direkte Pflege und Versorgung betreffend? - Den Personaleinsatz betreffend? - Den Einsatz von ehrenamtlich Aktiven betreffend? - Hygienevorgaben, Probleme in der Umsetzung? - Die Dokumentation/Kommunikation betreffend? |
| 4. | **Bitte erzählen Sie mir, wie die Einhaltung der Vorgaben zur Kohortierung/Isolierung bei Ihnen umgesetzt werden konnte.** | - Bewohner*innen - Mitarbeiter*innen |
| 5. | **Wie haben sich die Maßnahmen zur Kontaktbeschränkung auf die sozialen Aktivitäten, wie gemeinsames Essen, Veranstaltungen oder Gruppenangebote innerhalb ihres Wohnbereichs ausgewirkt?** | - Einzel- /Gruppenangebote? - Gemeinsames Kochen/Singen etc.? |
| 6. | **Bitte erzählen Sie mir von Maßnahmen, mit denen Sie versucht haben und aktuell versuchen, die soziale Teilhabe und die Lebensqualität der Bewohner*innen aufrechtzuerhalten.** | - Digitale Lösungen? - Besuchsregelungen für Ärzt*innen, Therapeut*innen, Fußpflege, Angehörige? |
| 7. | **Bitte erzählen Sie mir, wie Sie mit den unterschiedlichen Beteiligten über die Pandemie und die daraus resultierenden Maßnahmen kommuniziert haben.** | - Bewohner*innen? - Mitarbeiter*innen? - Angehörige, gesetzliche Betreuer*innen? - Ärzt*innen, Therapeut*innen, etc. ? |
| 8. | **Wir gehen davon aus, dass die Pandemie mit einem erheblichen Mehraufwand für Sie verbunden war. Bitte erzählen Sie mir, wie das genau war.** | - Mehraufwand im Personalmanagement? - Schulung von Mitarbeiter*innen? - In der Kommunikation mit Angehörigen? - In der Kommunikation mit externen Akteuren (z.B. Gesundheitsamt, Ärzt*innen, Therapeut*innen?) |
| 9. | **Bitte erzählen Sie mir, was die Pandemie und deren Folgen aus Ihrer Sicht für die Bewohner*innen und deren Angehörige bedeutet?** | - Soziale Isolation? Unsicherheit, Ängste? - Gesundheit? - Schutz? |
| 10. | **Bitte erzählen Sie mir, was die Pandemie und deren Folgen aus Ihrer Sicht für Ihre Mitarbeiter*innen (Wohnbereichsteam) bedeutet?** |  |
| 11. | **Gibt es aktuell ein Thema, das Ihnen Probleme oder Sorgen im Zusammenhang mit der direkten Pflege und der COVID-19 Pandemie bereitet?** |  |
| 12. | **Wo sehen Sie dringenden Unterstützungsbedarf im Zusammenhang mit der COVID-19 Pandemie für Ihren Wohnbereich?** |  |
| **Überleitung zum Themenkomplex Gewalt und Gewaltprävention:**  **Wir können uns vorstellen, dass die COVID-19-Krise zu Stress und vielleicht auch Spannungen zwischen Pflegekräften und Bewohner*innen führen kann, die nicht immer gut zu lösen sind.** | | |
| 13. | Wenn Sie an die aktuellen Herausforderungen der COVID-19 Krise denken und an die Auswirkungen auf die Bewohner*innen und Mitarbeiter*innen in ihrer Einrichtung, welche Bedeutung hat für Sie das Thema Prävention von Gewalt? |  |
| 14. | **Auswirkung der COVID-19 Pandemie auf das Gewaltgeschehen in den Einrichtungen** | |
|  | - Inwiefern hat sich nach Ihrer Einschätzung das Gewaltgeschehen in Ihrer Einrichtung durch die aktuellen Herausforderungen der Pandemie verändert? - Erzählen Sie bitte von einer Situation im Zusammenhang mit Gewalt, in der Sie oder Ihre Kolleg*innen dachten, das wäre ohne COVID-19 nicht passiert? |  |
| 15. | **Maßnahmen zur Gewaltprävention** | |
|  | - Welche Maßnahmen zur Gewaltprävention werden in Ihrer Einrichtung generell umgesetzt? - Welche Maßnahmen würden Sie als sinnvoll erachten, um dem Thema Gewalt in der aktuellen Situation zu begegnen? |  |
| 16. | **Umsetzbarkeit der Maßnahmen zur Gewaltprävention unter den aktuellen Herausforderungen der COVID19 Pandemie** | |
|  | - Was denken Sie, wie gut sind die Maßnahmen zur Gewaltprävention unter den aktuellen Herausforderungen [der COVID-19 Pandemie] umsetzbar? |  |
| **Ergänzende Frage für PEKo teilnehmende Einrichtungen:** | | |
| 17. | **Profit/ Mehrwert durch die Teilnahme am Projekt PEKo bzw. die geplanten und umgesetzten Maßnahmen zur Gewaltprävention** | |
|  | - Erzählen Sie bitte von einer Situation im Zusammenhang mit COVID-19, in der Sie dachten, gut, dass wir bei PEKo mitgemacht haben? - Wenn Sie sich zurückerinnern und vergleichen: Sind Sie aus Ihrer Sicht in der aktuellen Situation durch die im PEKo-Projekt geplanten und umgesetzten Maßnahmen zur Gewaltprävention besser aufgestellt? |  |

| **Merkmale, die durch das Studienzentrum dokumentiert werden** | | |
| --- | --- | --- |
|  | **Interview Nummer:** |  |
| 1. . | **Verbindung zur Projektgruppe**   - PEKo - Anderes laufendes Projekt:   Anderes geplantes Projekt: |  |
|  | **In welchem Bundesland liegt Ihre Einrichtung:** |  |
|  | **Ist die Ansiedlung Ihrer Einrichtung eher**   - Ländlich - Städtisch |  |
| **Merkmale der stationären Altenpflegeeinrichtungen sowie der Interviewteilnehmer*in**   - Fragen 5 bis 7 werden von allen Interviewteilnehmer*innen beantwortet - Fragen 8 bis 20 werden nur von Einrichtungsleitungen beantwortet - Frage 21 wird nur von Wohnbereichsleitungen beantwortet | | |
| **Nr.** | **Thema/Fragen/Antworten** |  |
|  | **In welcher Funktion sind Sie in der Einrichtung tätig?**   - Einrichtungsleitung - Pflegedienstleitung - Wohnbereichsleitung - Sonstiges |  |
|  | **Abfrage von soziodemografischen Charakteristika**   - Berufserfahrung in Jahren in der stationären Altenpflege (inkl. Ausbildung):   *(Angabe in ganzen Jahren)*   - Berufserfahrung in Jahren in der aktuellen Position (unabhängig von der Einrichtung):   *(Angabe in ganzen Jahren)* |  |
|  | **Erfahrung in der beruflichen Position mit Infektionskrankheiten, die erweiterte Maßnahmen erforderten (z.B. Norovirus-Infektion, Influenza, Multiresistente Erreger)?**   - Ja - Nein |  |
|  | **In welcher Trägerschaft befindet sich Ihre Einrichtung?**   - Öffentlich - Freigemeinnützig - Privat |  |
|  | **Hat Ihre Einrichtung einen pflegefachlichen Schwerpunkt (laut Versorgungsvertrag)**   - Demenz - Wachkoma - Beatmung - Gerontopsychiatrie - Multiple Sklerose - Psychische Erkrankungen - Geistige Behinderungen - Junge Schwerstpflegebedürftige - Sonstiges: - Nein, kein Schwerpunkt laut Versorgungsvertrag |  |
|  | **Aktuelle Anzahl der Pflegeplätze in Ihrer Einrichtung (verfügbare Plätze)?** |  |
|  | **Gab oder gibt es bestätigte COVID-19 Infektionen unter Ihren Bewohner*innen?**   - Nein - Ja - Wenn ja, wie viele? |  |
|  | **Gab oder gibt es Verdachtsfälle (ohne bekanntes Testergebnis) unter Ihren Bewohner*innen?**   - Nein - Ja - Wenn ja, wie viele? |  |
|  | **Gab es bestätigte COVID-19 Todesfälle unter den Bewohner*innen in Ihrer Einrichtung?**   - Nein - Ja - Wenn ja, wie viele? |  |
|  | **Wie viele Ihrer Bewohner*innen wurden bis heute insgesamt auf COVID-19 getestet?**  Anzahl: |  |
|  | **Wie viele Bewohner*innen sind bis heute aufgrund einer bestätigten COVID-19 Diagnose in ein Krankenhaus verlegt worden?**  Anzahl: |  |
|  | **Gab oder gibt es bestätigte COVID-19-Infektionen unter Ihren Mitarbeiter*innen?**   - Nein - Ja - Wenn ja, wie viele? |  |
|  | **Gab oder gibt es Verdachtsfälle (ohne bekanntes Testergebnis) unter Ihren Mitarbeiter*innen?**   - Nein - Ja - Wenn ja, wie viele? |  |
|  | **Wie viele Ihrer Mitarbeiter*innen wurden bis heute insgesamt auf COVID-19 getestet?**  Anzahl: |  |
|  | **Wie viele Ihrer Mitarbeiter*innen wurden bis heute aufgrund einer bestätigten COVID-19 Diagnose arbeitsunfähig gemeldet?**  Anzahl: |  |
|  | **Wie viele Mitarbeiter*innen Ihrer Einrichtung sind bis heute aufgrund einer bestätigten COVID-19 Diagnose in einem Krankenhaus behandelt worden?**  Anzahl: |  |
|  | **Welches Wohnkonzept wird auf Ihrem Wohnbereich umgesetzt?**   - Wohngruppe/Hausgemeinschaft - Klassischer Wohnbereich - Sonstiges: |  |
